# The epidemiology and detectability of asymptomatic *plasmodium vivax* and *plasmodium falciparum* infections in low, moderate and high transmission settings in Ethiopia

**DOI:** 10.1186/s12936-021-03587-4

**Published:** 2021-01-22

**Authors:** Elifaged Hailemeskel, Surafel K Tebeje, Sinknesh W. Behaksra, Girma Shumie, Getasew Shitaye, Migbaru Keffale, Wakweya Chali, Abrham Gashaw, Temesgen Ashine, Chris Drakeley, Teun Bousema, Endalamaw Gadisa, Fitsum G. Tadesse

**Affiliations:** 1 Malaria and Neglected Tropical Diseases Directorate, Armauer Hansen Research Institute, PO Box 1005, Addis Ababa, Ethiopia; 2 Department of Biomedical Sciences, College of Natural and Computational Sciences, Addis Ababa University, PO Box 1176, Addis Ababa, Ethiopia; 3 Department of Biology, College of Natural and Computational Sciences, Wollo University, PO Box, 1145, Dessie, Ethiopia; 4 Department of Medical Microbiology, Radboud University Medical Center, 6525 GA Nijmegen, The Netherlands; 5 Institute of Biotechnology, Addis Ababa University, PO Box, 1176 Addis Ababa, Ethiopia; 6 Department of Biomedical Sciences, School of Medical Sciences, Bahir Dar University, Bahir Dar, Ethiopia; 7 Department of Immunology and Infection, London School of Hygiene & Tropical Medicine, WC1E 7HT London, UK

**Keywords:** *Plasmodium* infection, Elimination, Asymptomatic, Transmission, nPCR, Detectability, Density distribution

## Abstract

**Background:**

As countries move to malaria elimination, detecting and targeting asymptomatic malaria infections might be needed. Here, the epidemiology and detectability of asymptomatic *Plasmodium falciparum* and *Plasmodium vivax* infections were investigated in different transmission settings in Ethiopia.

**Method:**

A total of 1093 dried blood spot (DBS) samples were collected from afebrile and apparently healthy individuals across ten study sites in Ethiopia from 2016 to 2020. Of these, 862 were from community and 231 from school based cross-sectional surveys. Malaria infection status was determined by microscopy or rapid diagnostics tests (RDT) and 18S rRNA-based nested PCR (nPCR). The annual parasite index (API) was used to classify endemicity as low (API > 0 and < 5), moderate (API ≥ 5 and < 100) and high transmission (API ≥ 100) and detectability of infections was assessed in these settings.

**Results:**

In community surveys, the overall prevalence of asymptomatic *Plasmodium* infections by microscopy/RDT, nPCR and all methods combined was 12.2% (105/860), 21.6% (183/846) and 24.1% (208/862), respectively. The proportion of nPCR positive infections that was detectable by microscopy/RDT was 48.7% (73/150) for *P. falciparum* and 4.6% (2/44) for *P. vivax*. Compared to low transmission settings, the likelihood of detecting infections by microscopy/RDT was increased in moderate (Adjusted odds ratio [AOR]: 3.4; 95% confidence interval [95% CI] 1.6–7.2, P = 0.002) and high endemic settings (AOR = 5.1; 95% CI 2.6–9.9, P < 0.001). After adjustment for site and correlation between observations from the same survey, the likelihood of detecting asymptomatic infections by microscopy/RDT (AOR per year increase = 0.95, 95% CI 0.9–1.0, P = 0.013) declined with age.

**Conclusions:**

Conventional diagnostics missed nearly half of the asymptomatic *Plasmodium* reservoir detected by nPCR. The detectability of infections was particularly low in older age groups and low transmission settings. These findings highlight the need for sensitive diagnostic tools to detect the entire parasite reservoir and potential infection transmitters.

## Introduction

Following considerable successes in the control of malaria in the last two decades, progress plateaued or stalled in many settings in Africa [1]. Ethiopia runs a successful malaria control programme [2] that makes it one of the four countries (together with India, Rwanda, and Pakistan) that continues to maintain the declining trend in malaria burden [3]. As a result, the country is on track for a 40% reduction in incidence (together with Rwanda, Zambia, and Zimbabwe) and malaria mortality rates (together with Zambia) by 2020 [1]. To guide elimination efforts that currently targets 239 selected districts, the National Malaria Control Programme (NMCP) of Ethiopia stratified the country into four strata using district level annual parasite index (API) data from 2017 [4] as malaria-free (API, 0), low (API, 0–5), moderate (API, 5–100), and high (API, ≥ 100) [4]. Despite its value, the adopted stratification lacks granularity and is not able to capture relevant spatial and temporal heterogeneities in low endemic settings [5, 6]. The unique epidemiology of malaria transmission in Ethiopia; the presence of strictly seasonal transmission in some settings and perennial transmission elsewhere, as well as different levels of co-endemicity of *Plasmodium falciparum* and *Plasmodium vivax* [2], calls for the use of tailored approaches to characterize the epidemiology of malaria.

District level stratification that relies on malaria incidence data has limitations in settings where case numbers are extremely low. Incidence data are also sensitive to changes in care seeking behavior, rates of testing of suspected cases, and reporting completeness [7]. Screening approaches to determine the prevalence of (often asymptomatic) infections that are present in communities have great potential to define transmission intensity [8]. However, parasite prevalence estimates are greatly affected by parasite density distributions in communities that determine the detectability of infections by different diagnostics. Malaria elimination efforts may benefit from targeting all infections present in communities, irrespective of clinical presentation [9–11]. There is a growing body of evidence on the public health importance of asymptomatic malaria infections and their contribution to onwards malaria transmission in high [12, 13] and low transmission settings [13, 14]. Importantly, most asymptomatic infections detected in community surveys are of low parasite density and the proportion of all infections that are submicroscopic varies between settings [15]. Previous studies in Ethiopia detected a significant burden of asymptomatic *P. falciparum* and *P. vivax* infections [16–19]. These studies used different diagnostic techniques and sampling designs, making it difficult to compare parasite prevalence estimates or diagnostic performance indicators across settings. The aim of the present study was to understand the epidemiology of asymptomatic *Plasmodium* infections in different settings in Ethiopia and their detectability by microscopy, rapid diagnostics test (RDT) and molecular methods.

## Methods and materials

### Study areas

**Fig. 1:**
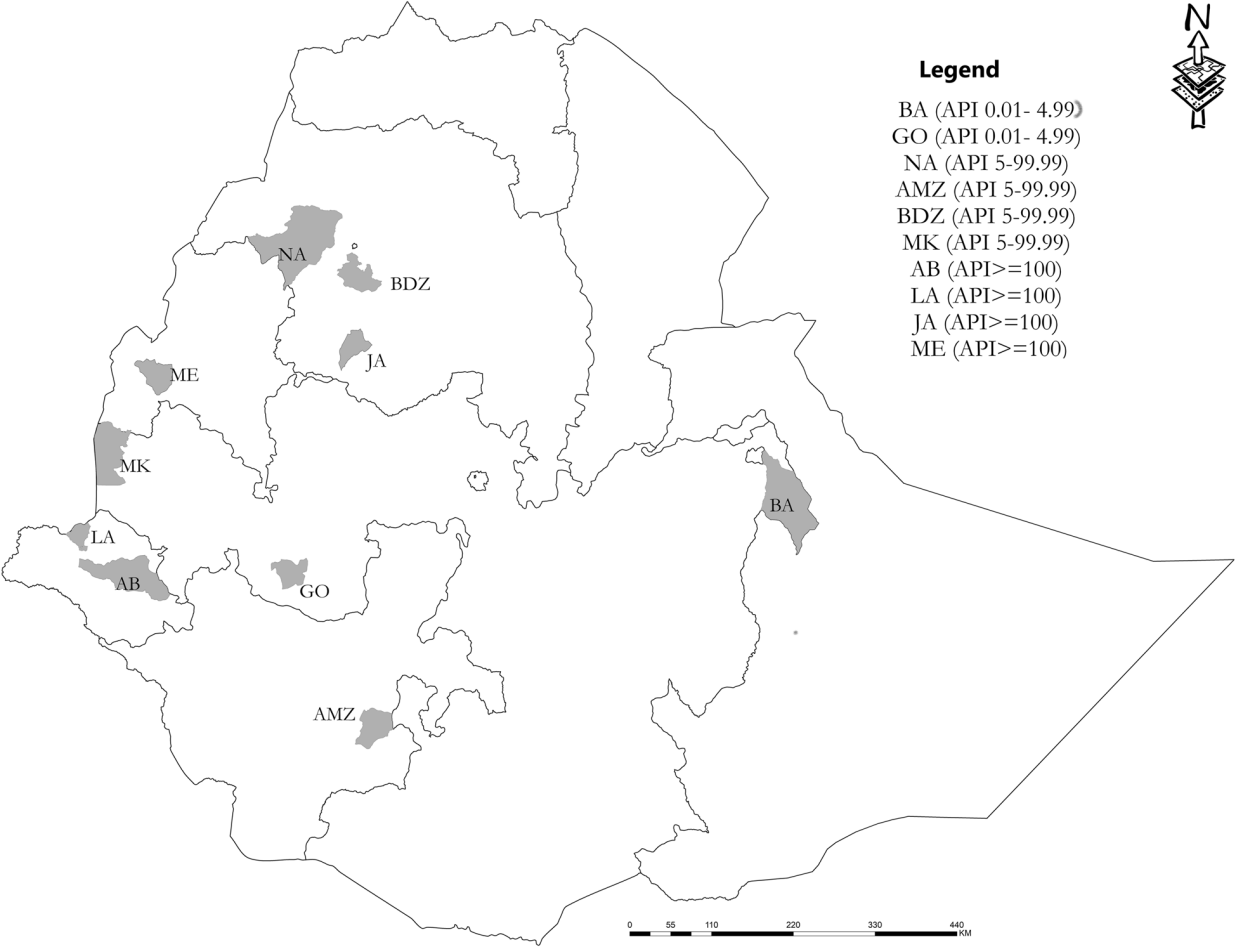
Location of study sites and their Annual Parasite Index (API) as per the stratification by National Malaria Control Program based on 2017 data: BA = Babile, GO = Gomma, NA = North Achefer, AMZ = ArbaMinch Zuria, BDZ = Bahir Dar Zuria, MK = Mao-komo, AB = Abobo, LA = Lare, JA = Jawi, ME = Meng

The study was conducted in ten districts (woredas) encompassing different transmission settings (Fig. 1). Malaria transmission is highly heterogeneous in Ethiopia and transmission intensity varies spatially and temporally [20]. Study sites representing low (n = 2), moderate (n = 4), and high (n = 4) transmission settings as per the national stratification were selected from five administrative regions (Fig. 1). Low transmission settings include Gomma and Babile districts from Oromia region. Moderate transmission settings include Bahir Dar Zuria and North Achefer districts from Amhara region and Arba Minch Zuria from the Southern region and Mao Komo from Benishangul region. High transmission districts were from Gambela (Lare and Abobo), Amhara (Jawi), and Benishangul (Meng) regions.

## Study population and sample collection

Samples were collected in community and school-based cross-sectional surveys from 2016 to 2020. Specifically, community-based surveys were conducted at Abobo, Lare, Mao-komo, Menge, and Gomma districts in 2016, Babile district in 2018, and Arba Minch Zuria district in 2020. School based surveys were conducted at North Achefer, Bahir Dar Zuria, and Jawi districts in 2017. For the school-based surveys, students were randomly selected from elementary school students stratified by age as described before [21] following protocols developed by Brooker and colleagues [22].

Prior to recruitment of participants for community surveys, sensitization was undertaken by teams that involve study team members, village-based health extension workers, malaria focal person of the district, local administrators, and elderly. The study purpose, procedure, risk, and benefit were explained in local language. After this first step, volunteer community members were invited to join the study upon obtaining informed written consent and enrolled in the study on first come, first served basis.

Finger prick blood samples (~ 300 µL) collected from all participants were used to diagnose malaria using RDT (First Response® malaria Antigen pLDH/HRP2 P.f and Pan Combo Card Test, Premier Medical Corporation Ltd, Dist. Valsad, India) or thin and thick blood films, and to prepare dried blood spots (DBS) on 3MM Whatman filter papers (Whatman, Maidstone, UK). Malaria was diagnosed using RDT at Abobo, Lare, Mao-Komo, Menge, and Gomma districts whilst microscopy was used at the school surveys, Arba Minch Zuria and Babile districts. Detailed clinical and socio-demographic data were captured using a pretested semi-structured interview-based questionnaire. Axillary body temperature was measured for all participants. If a participant was found febrile (axillary temperature ≥ 37.5 °C) or reports history of fever in the past 48 h, malaria status was checked using RDT and treated immediately when found positive following the national treatment guideline [23]. DBS were air dried, protected from direct sunlight, and enclosed in zip locked plastic bags individually with self-indicating silica gel (Loba Chemie, Mumbai, India). Samples were transported at ambient temperature and stored at − 20 °C until further use. Giemsa-stained thick and thin smears were read independently by two experienced malaria microscopists. A third expert microscopist was consulted in case of discordant results. Thick smear slides were declared negative if no parasites were detected after observing 100 fields under oil immersion (100× magnification).

### Species specific detection of ***Plasmodium*** parasites by 18S rRNA based nested polymerase chain reaction

Genomic DNA was extracted from 6 mm diameter DBS punches using Chelex-Saponin extraction method [24]. In brief, DNA was eluted after an overnight lysis in 0.5% saponin (SIGMA)/PBS (SIGMA) buffer and washing step followed by boiling at 97 ˚C in 150 µL of 6% Chelex (Bio Rad) in DNase/RNase free water (SIGMA). From the final eluate, 80 µL was transferred into a new plate and stored at − 20 ˚C until further use. *Plasmodium* species identification was done by nested polymerase chain reaction (nPCR) that targeted the small subunit 18S rRNA gene as described before [25]. A positive control (for *P. falciparum* NF54 culture from Radboudumc, Nijmegen, The Netherlands; for *P. vivax* the malaria reference laboratory positive controls from the London School of Hygiene and Tropical Medicine, London, UK) and negative controls (PCR grade water) were run in every reaction plate. Amplified products were visualized using UV transilluminator (Bio Rad, USA) after electrophoresis using 2% agarose gels (SIGMA, ALDRICH) stained with Ethidium Bromide (Promega, Madison, USA).

### Statistical analysis

For the school surveys, sample size was calculated based on protocols by Brooker and colleagues [22] for the original study that aimed at assessing longitudinal evaluation of parasite prevalence in school children [21]. For this study, 70.0% (231/330) of the students were successfully sampled. For the community surveys, an overall prevalence of 6.8% asymptomatic *Plasmodium* infections was expected based on previous observations [17, 19, 26–35] with a precision of 5%. Based on previous experience, a minimum of 75 samples for the school surveys and 114 for the community samples was targeted across the study sites [21]. Data was double entered into excel, compiled, checked for consistency, and analyzed using Stata version 15 (Stata corporation; College Station, TX, USA) and GraphPad Prism 5.3 (GraphPad Software Inc., CA, USA). Proportions were compared between categories using Fisher’s exact test and Pearson’s chi-squared test where it was appropriate. Equality tests on unmatched data such as age between school and community surveys were tested by two-sample Wilcoxon rank-sum (Mann-Whitney) test. Generalized Estimating Equation (GEE) was used to allow parameter estimates and standard errors adjusted for clustering across the study sites; exchangeable correlation matrix and robust standard errors were used. Sample characteristics such as age, gender, and transmission intensity were tested in the model for their association with infection prevalence and roles as potential confounders. A 5% level of significance was considered in all cases.

## Results

### Characteristics of study participants

A total of 1093 individuals, 231 from school (3 schools; 75–80 per school survey) and 862 from community surveys (7 surveys; 114–161 per study site) participated in the study. None of the partifcipants was febrile at the time of sampling. Female participants constituted 43.5% (372/855) of community and 51.8% (118/228) of school surveys (P = 0.026). The overall median age of the participants was 16 years (Interquartile range [IQR]: 11–35). As expected, participants from the school surveys were younger (median age, 12; IQR, 11–14) than community surveys (median age, 23; IQR, 10–38; P < 0.001). Results are presented separately for community and school surveys, focusing on community surveys for the main comparisons (Table 1). Within the community surveys, participants from low (median age, 30; IQR, 18–45; n = 232) and moderate (median age, 30; IQR, 12–42; n = 272) endemic settings were older than participants from high endemic settings (median age, 13; IQR, 8–28; n = 318; P < 0.001).

**Table 1:** Community-based prevalence of asymptomatic *Plasmodium* infection using nPCR and microscopy/RDT

Attributes	Category	Parasite prevalence by nPCR, % (n/N) [95% CI]	*P-value*	Parasite prevalence by microscopy/RDT, % (n/N) [95% CI]		*P-value*
Gender	Male	22.5 (106/472) [18.9–26.5]	0.442	13.5(65/483) [10.7–16.8]		0.232
	Female	20.2 (74/367) [16.4–24.6]		10.7(40/372) [7.9–14.3]		
Age group (years)	≤ 5	21.5 (14/65) [13.1–33.3]	0.008	10.6 (7/66) [5.1–20.7]		< 0.001
	5–15	26.7 (62/232) [21.4–32.8]		20.7 (50/241) [16.1–26.3]		
	≥ 15	16.9 (87/513) [13.9–20.5]		5.6 (29/515) [3.9–7.9]		
Study sites (n/N) High transmission	Lare	46.1 (47/102) [36.6–55.8]	< 0.001	35.9 (41/114) [27.6–45.2]		< 0.001
	Abobo	34.2 (39/114) [26.1–43.4]		23.7 (28/118) [16.9–32.3]		
	Meng	17.6 (21/119) [11.8–25.6]		10.1 (12/119) [5.8–16.9]		
Moderate transmission	Mamo-Komo	29.3 (34/116) [21.7–38.3]		16.4 (19/116) [10.7–24.3]		
	Arba Minch zuria	12.4 (20/161) [8.1–18.5]		1.8 (3/161) [0.6–5.6]		
Low transmission	Babile	15.4 (18/117) [9.9–23.2]		1.7 (2/117) [0.4–6.6]		
	Gomma	3.4 (4/117) [1.3–8.8]		0.0 (0/115 [NA]		
Transmission intensity	High	31.9 (107/335) [6.3–13.9]	< 0.001	23.1(81/351) [18.9–27.8]		< 0.001
	Moderate	19.5 (54/277) [15.2–24.6]		7.9 (22/277) [5.3–11.8]		
	Low	9.4 (22/234) [6.3–13.9]		0.9 (2/232) [0.2–3.4]		
Overall Prevalence (n/N)	–	21.6 (183/846) [18.9–24.5]		12.2 (105/860) [10.1–14.6]		

Age was missed for 40 samples. Gender was missed for seven samples

*CI* confidence interval, *API* Annual Parasite Index /1000 people

### Prevalence of asymptomatic malaria infection across the study sites

In the community surveys, the overall prevalence of asymptomatic *Plasmodium* infections was 12.2% (105/860) by microscopy/RDT and 21.6% (183/846) by nPCR (Table 1); 24.1% (208/862) of participants were parasite positive by either nPCR and/or microscopy/RDT. When considering infecting *Plasmodium* species by nPCR, 16.4% (139/846) of samples were *P. falciparum* positive; 3.7% (31/846) were *P. vivax* and 1.5% (13/846) were mixed *P. vivax* and *P. falciparum*. Although the school surveys were from high and moderate transmission sites, there was overall lower *Plasmodium* infection prevalence in the school surveys than in the community surveys as measured by all methods combined (11.3% vs. 24.1%; χ^2^ = 17.9, P < 0.001).

Among the school surveys, the overall prevalence of asymptomatic malaria was 0.4% (1/231) by microscopy/RDT whilst 11.3% (26/231) were parasite positive either by nPCR or both methods combined. Of these nPCR positive malaria infections from the school surveys, 2.6% (6/231) were due to *P. falciparum*, 5.2% (12/231) were due to *P. vivax*, and 3.5% (8/231) were due to mixed *P. falciparum* and *P. vivax* species infections (Additional file 1: Table S1, Additional file 2: Table S2).

Across the community surveys, in high transmission settings, nPCR-based prevalence of malaria infection ranged from 17.6% (n/N) at Meng to 46.1% (47/102) at Lare district. In the moderate transmission sites, the nPCR-based prevalence was 29.3% (34/116) at Mao-Komo and 12.4% (20/161) at Arbaminch Zuria district. In low transmission sites, the overall nPCR infection prevalence was 9.4% (22/234). The overall microscopy/RDT based prevalence was 23.1% (81/351), 7.9% (22/277), and 0.9% (2/232), in high, moderate, and low transmission settings, respectively (Table 1).

Among the community samples, the prevalence of *Plasmodium* infections detected by all methods combined was substantially higher for the high transmission settings (36.7%, 129/351; 95% CI 31.9–41.9; P < 0.001) compared to moderate (20.6%, 57/277; 95% CI 16.2–25.8) and low transmission settings (9.4%, 22/234; 95% CI 6.3–13.9). Moreover, the burden of asymptomatic *Plasmodium* infection was higher in the 5–15 age groups as measured by microscopy/RDT (20.7%, 50/241, P < 0.001) and nPCR (26.7%, 62/232, P = 0.008) (Table 1) as compared to under-five children and adults older than 15 years (Table 1).

### Detectability of asymptomatic *Plasmodium* infections in different endemicities

**Fig. 2:**
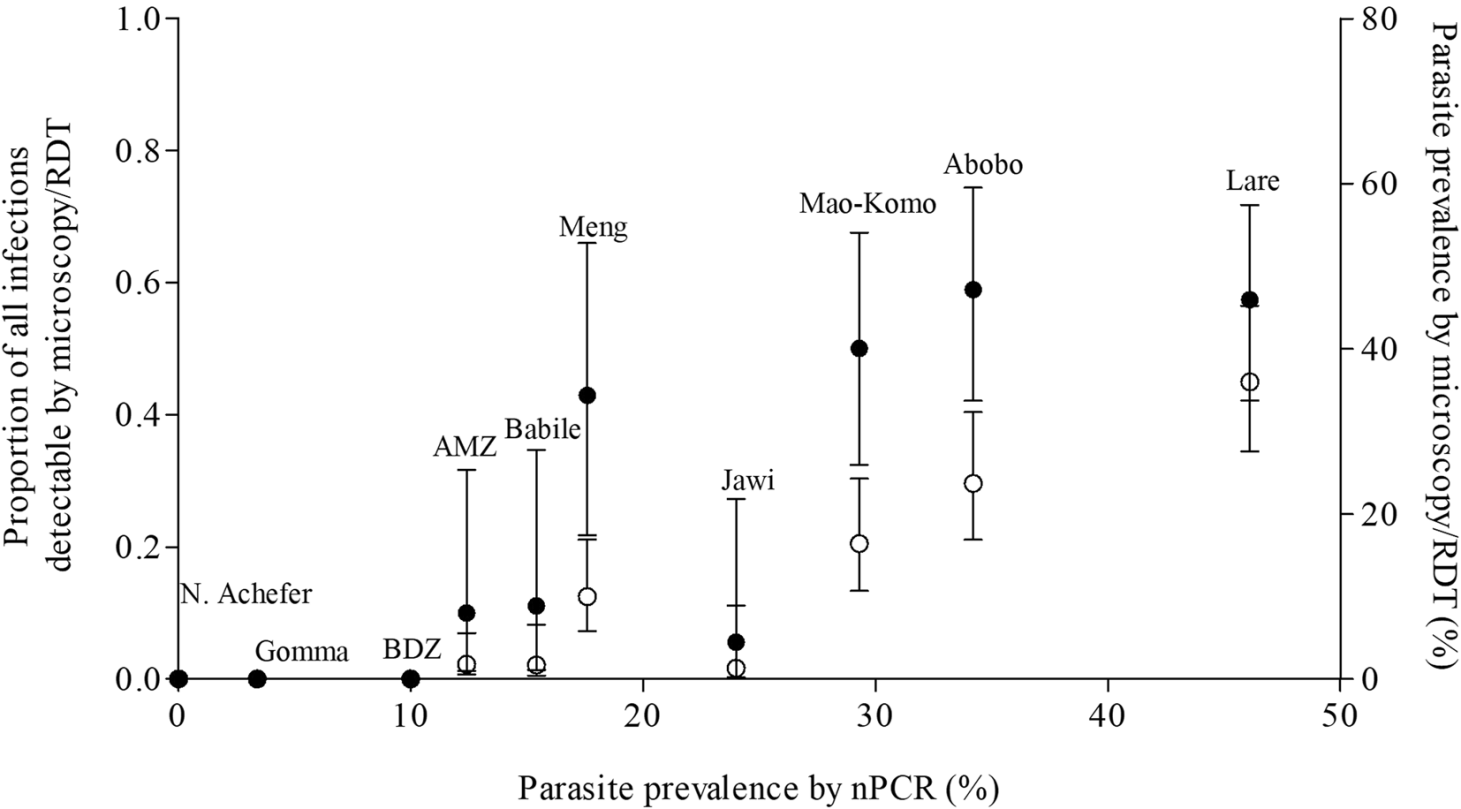
Community and school- based surveys asymptomatic malaria infection prevalence and detectability using nPCR and microscopy/RDT: black circles represent parasite prevalence by nPCR (x-axis) and proportion all infections detected by microscopy/RDT (left y-axis); white circles indicate parasite prevalence by microscopy/RDT (right y-axis). School surveys were (N. Achefer) North Achefer, BDZ (Bahir Dar Zuria) and Jawi

Among community samples, microscopy/RDT detected 44.2% (80/181) of nPCR detected *Plasmodium* infections (Agreement = 86.9%, κ = 0.526, Table 2). All, but 8 RDT positive *P. falciparum* and 1 microscopy positive *P. vivax* sample, were also nPCR positive (Additional file 2: Table S2). The likelihood that *Plasmodium* infected individuals (i.e. individuals who were parasite positive by any diagnostic method) were detected by RDT was increased for individuals living in higher transmission settings (AOR = 5.1, 95% CI 2.6–9.9, P < 0.001) and individuals living in moderate transmission (AOR: 3.4, 95% CI 1.6–7.2, P = 0.002) compared to low transmission settings (Additional file 3: Table S3; Fig. 2). Age was an important predictor of asymptomatic malaria positivity by microscopy/RDT. After adjusting for site and correlation between observations from the same survey, a 5% decline in detection using microscopy/RDT was observed for every year increase of age from those that tested positive by all methods (AOR = 0.95, 95% CI 0.9–1.0, P = 0.013).

The parasite species composition and detectability varied between transmission settings (Fig. 2). Among the *Plasmodium* species detected in the community samples, the majority were attributable to *P. falciparum* (77.4%, 161/208) when all samples were combined. Of the nPCR detected *P. falciparum-*mono species infections (n = 139) and mixed-species infections (n = 13), microscopy/RDT successfully detected *P. falciparum* in 48.7% (73/150) of infections (Table 2). Of the nPCR detected *P. vivax-*mono infections (n = 31) and mixed-species infections (n = 13), microscopy/RDT successfully detected *P. vivax* in 4.6% (2/44) of infections (Table 2).

**Table 2:** Species specific asymptomatic malaria infection prevalence and detectability across the study sites using nPCR and microscopy/RDT from 2016-2020

Sample source and API	Study sites	Microscopy/RDT % [n/N]				nPCR % [n/N]				Proportion of infections detected by microscopy/RDT (%)		
		Pf	Pv	Mixed	Proportion (%) of Pv	Pf	Pv	Mixed	Proportion (%) of Pv	Any	Pf	Pv
Community- (API ≥ 100)	Lare*	33.3 [38/114]	0.0 [0/114]	2.6 [3/114]	7.3 [3/41]	41.2 [42/102]	0.9 [1/102]	3.9 [4/102]	10.6 [5/47]	57.4 [27/47]	56.5 [26/46]	0.00 [0/5]
	Abobo¥	17.8 [21/118]	0.0 [0/118]	5.9 [7/118]	25.0 [7/28]	28.1 [32/114]	3.5 [4/114]	2.6 [3/114]	17.9 [7/39]	58.9 [23/39]	57.1 [20/35]	14.3 [1/7]
	Meng	10.1 [12/119]	0.0 [0/119]	0.0 [0/119]	0.0 [0/12]	13.5 [16/119]	3.4 [4/119]	0.8 [1/119]	23.8 [5/21]	42.9 [9/21]	47.1 [8/17]	0.0 [0/5]
	Total	20.2 [71/351]	0.0 [0/351]	2.8 [10/351]	12.3 [10/81]	26.9 [90/335]	2.7 [9/335]	2.4 [8/335]	15.9 [17/107]	55.1 [59/107]	55.1 [54/98]	5.8 [1/17]
Community – (API ≥ 5&<100)	Mao-komo	16.4 [19/116]	0.0 [0/116]	0.0 [0/116]	0.0 [0/19]	18.9 [22/116]	9.5 [11/116]	0.8 [1/116]	35.3 [12/34]	50.0 [17/34]	65.2 [15/23]	0.0 [0/12]
	AMZ	0.0 [0/161]	0.6 [1/161]	1.2 [2/161]	50.0 [3/6]	6.8 [11/161]	3.1 [5/161]	2.5 [4/161]	45.0 [9/20]	10.0 [2/20]	13.3 [2/15]	11.1 [1/9]
	Total	6.8 [19/277]	0.4 [1/277]	0.72 [2/277]	13.6 [3/22]	11.9 [33/277]	5.8 [16/277]	1.8 [5/277]	38.9 [21/54]	35.2 [19/54]	44.7 [17/38]	4.8 [1/21]
Low (API > 0 and < 5)	Gomma§	0.00 [0/115]	0.0 [0/115]	0.0 [0/115]	0.0 [0/115]	2.6 [3/117]	0.8 [1/117]	0.0 [0/117]	25.0 [1/4]	0.0 [0/2]	0.0 [0/1]	0.0 [0/1]
	Babile	1.7 [2/117]	0.0 [0/117]	0.0 [0/117]	0.0 [0/2]	11.1 [13/117]	4.3 [5/117]	0.0 [0/117]	27.8 [5/18]	15.4 [2/18]	15.4 [2/13]	0.0 [0/5]
	Total	0.9 [2/232]	0.0 [0/232	0.0 [0/232]	0.0 [0/2]	6.8 [16/234]	2.6 [6/234]	0.0 [0/234]	27.3 [6/22]	10.0 [2/20]	14.3 [2/14]	0.0 [0/6]
Community	Grand Total	10.7 [92/860]	0.1 [1/860]	1.4 [12/860]	12.4 [13/105]	16.4 [139/846]	3.7 [31/846]	1.5 [13/846]	24.0 [44/183]	44.2 [80/181]	48.7 [73/150]	4.6 [2/44]
School (API ≥ 100)	Jawi	1.3 [1/75]	0.0 [0/75]	0.0 [0/75]	0.0 [0/1]	5.3 [4/75]	10.7 [8/75]	8.00 [6/75]	77.8 [14/18]	5.6 [1/18]	10.0 [1/10]	0.0 [0/14]
School (API ≥ 5 and < 100)	BDZ	0.0 [0/80]	0.0 [0/80]	0.0 [0/80]	0.0 [0/80]	2.50 [2/80]	5.00 [4/80]	2.5 [2/80]	75.0 [6/8]	0.0 [0/8]	0.0 [0/4]	0.0 [0/6]
	N. Achefer	0.0 [0/76]	0.0 [0/76]	0.0 [0/76]	0.0 NA	0.0 [0/76]	0.0 [0/76]	0.0 NA	0.0 [0/76]	0.0 NA	0.0 NA	0.0 NA
School	Total	0.4 [1/231]	0.0 [0/231]	0.0 [0/231]	0.0 [0/1]	2.6 [6/231]	5.2 [12/231]	3.5 [8/231]	76.9 [20/26]	3.8 [1/26]	7.1 [1/14]	0.0 [0/20]

AMZ = Arba Minch Zuria, BDZ = Bahir Dar Zuria, N. Achefer = North Achefer, Pf = *P. falciparum*, Pv = *P. vivax*, mixed = Pf + Pv, Any = all species detected. Proportion of Pv is calculated together with mixed infections. API = annual parasite index /1000 people. * 12 DBS samples (11 Pf and 1 mixed infection positive by RDT) were missed from Lare for PCR. ^¥^ 4 DBS samples (3 Pf and 1 mixed infection positive by RDT) were missed from Abobo for PCR. ^§^ microscopy was not done for 2 samples that were Pf positive by PCR

## Discussion

This study describes the prevalence and detectability of asymptomatic *Plasmodium* infections in ten different transmission settings by nPCR and conventional diagnostics (i.e. microscopy/RDT). More asymptomatic infections were detected in high transmission settings by both methods. The detectability of asymptomatic *Plasmodium* infections using microscopy/RDT relative to nPCR increased as transmission intensity increases. As a result, most infections in low transmission settings were not detectable by microscopy/RDT.

In Ethiopia, several cross-sectional studies have documented asymptomatic parasite carriage using conventional and molecular methods [16–18, 33, 34]. The current multi-site study allowed an assessment of factors influencing the prevalence of infections as well as their detectability by microscopy-RDT. The prevalence of asymptomatic *Plasmodium* infections in the current study was in the same range as other reports from high [18, 34] and moderate [27] transmission settings in Ethiopia and elsewhere [17, 29, 36, 37].

Consistent with other studies [16, 38, 39], the current study observed that microscopy/RDT detected fewer asymptomatic infections as compared to PCR. The proportion of *Plasmodium* infections that was detectable by microscopy/RDT increased with increasing in transmission intensity. Whilst this trend has been reported in meta-analyses for *P. falciparum* [15, 36, 40], it is striking that this trend is also apparent in the current study within one country affected by both *P. falciparum* and *P. vivax*. Moreover, the effect size was comparatively large with approximately 5-fold higher detectability of infections in high endemic settings compared to low endemic settings. The trend of increasing detectability with increasing transmission intensity may be attributable to the fact that asymptomatically infected individuals have higher average parasite densities in high transmission settings [15, 41]. Moreover, in low endemic settings individuals will receive fewer infectious bites with, due to the absence of super-infections, lower parasitemia over the course of infection [9, 36]. Low genetic diversity of the parasite population in low transmission settings may also contribute to rapidly acquired immunity to the specific clones [42], further limiting parasite density. An impact of immunity on parasite density and the detectability of infections is also illustrated by the negative impact of increasing age on the detectability on infections in line with the current study [43].

Lower parasite densities in *P. vivax* compared to *P. falciparum* [44, 45] also results in a low detectability of *P. vivax* infections by microscopy/RDT. This low density in *P. vivax* is mainly attributable to the parasite’s preference to infect reticulocytes [46, 47] that typically constitute less than 1% of the total erythrocyte population [48] and also to the early acquisition of immunity [47]. These findings have implications for estimates of the relative burden of *P. falciparum* and *P. vivax* infections. The introduction of sensitive molecular tools may thus improve the detection of *P. vivax* infections substantially. Since treatment strategies differ for *P. falciparum* and *P. vivax*, this is relevant for public health interventions.

Although RDT and microscopy were used separately in the study sites due to logistics reasons, the prevalence measured by conventional RDT and microscopy was assumed to be comparable [37].

Nine samples that were declared microscopy/RDT positive were negative by nPCR while seven samples that were detected *P. falciparum* positive by RDT were *P. vivax* positive by nPCR. False RDT positivity might be due to the presence of parasite antigens after adequate clearance of parasites which might explain the variation between RDT positivity and PCR negative detection among asymptomatic malaria infections [49, 50]. Hence, there is a possibility that RDT can be positive for lingering antigens of *P. falciparum* while missing the low-density *P. vivax* infection from the same patient.

## Conclusions

Conventional diagnostics missed nearly half of the asymptomatic malaria reservoir detected by nPCR. Moreover, the detectability of asymptomatic *Plasmodium* infections in all endemic sites might reflect the long persistence of these infections from weeks up to months in high [51] as well as in low transmission settings [52, 53] even in the presence of effective control and elimination interventions. As these infections can have relevance for onward malaria transmission [13–15], a detailed understanding of the distribution, detectability, and contribution to the infectious reservoir of asymptomatic infections will greatly improve our ability to target all relevant infections. The wide scale presence of low-density infections calls for more in-depth studies on understanding parasite density oscillations, their relevance for malaria symptoms, and onward transmission to mosquitoes.

## Supplementary Information

**Additional file 1: Table S1.** School based prevalence of asymptomatic malaria in selected sites from different transmission settings using nPCR and microscopy/RDT from 2016 to 2018, Ethiopia.

**Additional file 2: Table S2.** Concordance of RDT and Microscopy detected samples compared to nPCR among the study participants, 2016–2020.

**Additional file 3: Table S3.** GEE model for association of malaria infection prevalence using all methods combined (nPCR and/or microscopy/RDT) among community survey samples with sample characteristics such as gender, age category, level of endemicity from 2016 to 2020, Ethiopia.

## Availability of data and materials

The datasets used and/or analyzed during the current study are available from the corresponding author on reasonable request.
