# Supplementary material for: The epidemiology and detectability of asymptomatic plasmodium vivax and plasmodium falciparum infections in low, moderate and high transmission settings in Ethiopia
Source: Malar J. 2021 Jan 22;20:59. doi: 10.1186/s12936-021-03587-4 (PMC7821398; doi:10.1186/s12936-021-03587-4)
Supplement: Supplementary file 1 — Additional file 1: Table S1. School based prevalence of asymptomatic malaria in selected sites from different transmission settings using nPCR and microscopy/RDT from 2016 to 2018, Ethiopia. [file 12936_2021_3587_MOESM1_ESM.docx]

| Attributes | Category | Parasite prevalence  by nPCR,  % (n/N) [95% CI] | *P-value* | Parasite prevalence  by microscopy/RDT,  % (n/N) [95% CI] | *P-value* | |
| --- | --- | --- | --- | --- | --- | --- |
|  |  | (N= 231) | 0. 544 | (N= 231) |  |  |
| Gender | Male | 12.7(14/110) [7.6-20.4] |  | 0.0 (0/110) [NA] | 0.333 |  |
|  | Female | 10.2(12/118) [5.8-17.1] |  | 0.85 (1/118) [0.01-5.8] |  |  |
| Age group (years) | <5 | - | 0.182 | - | 0.755 |  |
|  | 5-15 | 10.2 (21/206) [6.7-15.2] |  | 0.5 (1/206) [0.07-3.4] |  |  |
|  | >15 | 20.0 (4/20) [7.5-43.6] |  | 0.0 (0/20) [NA] |  |  |
| Study sites (n/N) |  |  |  |  |  |  |
| High transmission (API ≥100) | Jawi | 24.0 (18/75) [15.62-35.01] | <0.001 | 1.3 (1/75) [0.18-8.98] | 0.352 |  |
| Moderate transmission  (API≥5&<100) | BDZ | 10.0 (8/80) [5.05-18.83] |  | 0.0 (0/80) [NA] |  |  |
|  | N. Achefer | 0.0 (0/76) [NA] |  | 0.0 (0/76) [NA] |  |  |
| Overall Prevalence (n/N) | ------- | 11.3 (26/231) [7.5-16.0] |  | 0.4(1/231) [0.01-2.3] |  |  |

**Supplement Table 1**. School-based prevalence of asymptomatic malaria in selected sites from different transmission settings using nPCR and microscopy/RDT from 2016- 2018, Ethiopia
