# Supplementary material for: The epidemiology and detectability of asymptomatic plasmodium vivax and plasmodium falciparum infections in low, moderate and high transmission settings in Ethiopia
Source: Malar J. 2021 Jan 22;20:59. doi: 10.1186/s12936-021-03587-4 (PMC7821398; doi:10.1186/s12936-021-03587-4)
Supplement: Supplementary file 2 — Additional file 2: Table S2. Concordance of RDT and Microscopy detected samples compared to nPCR among the study participants, 2016–2020. [file 12936_2021_3587_MOESM2_ESM.docx]

**Supplementary Table-2.** Concordance of RDT and Microscopy detected samples compared to nPCR among the study participants, 2016-2020

|  |  | **18S nPCR** | | | |
| --- | --- | --- | --- | --- | --- |
|  |  | *P. falciparum* | | *P. vivax* | |
|  |  | Positive | Negative | Positive | Negative |
| **RDT** | Positive | 68 | 8 | 8 | 0 |
|  | Negative | 52 | 302 | 20 | 302 |
| **Microscopy** | Positive | 4 | 0 | 1 | 1 |
|  | Negative | 38 | 557 | 34 | 557 |

**NB:** the mixed species infections are added with the *P. falciparum* and *P. vivax* infections
