# Supplementary material for: The epidemiology and detectability of asymptomatic plasmodium vivax and plasmodium falciparum infections in low, moderate and high transmission settings in Ethiopia
Source: Malar J. 2021 Jan 22;20:59. doi: 10.1186/s12936-021-03587-4 (PMC7821398; doi:10.1186/s12936-021-03587-4)
Supplement: Supplementary file 3 — Additional file 3: Table S3. GEE model for association of malaria infection prevalence using all methods combined (nPCR and/or microscopy/RDT) among community survey samples with sample characteristics such as gender, age category, level of endemicity from 2016 to 2020, Ethiopia. [file 12936_2021_3587_MOESM3_ESM.docx]

**Supplementary Table 3.** GEE model for association of malaria infection prevalence using all methods combined (nPCR and/or microscopy/RDT) among community survey samples with sample characteristics such as gender, age category, level of endemicity from 2016-2020, Ethiopia

| Sample characteristics | | Total malaria infection prevalence | | | |
| --- | --- | --- | --- | --- | --- |
|  |  | COR  (95% CI) | P- value | AOR  (95% CI) | P-value |
| Gender | Female | 1 (Ref) |  | 1(Ref) | - |
|  | Male | 1.2  (0.8-2.0) | 0.385 | - | - |
| Age as continuous variable (years) |  | 0.95(0.9-1.0) | 0.001 | 0.95(0.9-1.0) | 0.013 |
| Level of endemicity | Low  (API >0 &<5) | 1(Ref) | - | 1(Ref) | - |
|  | Moderate  (API ≥5 & <100) | 5.5  (1.6-19.1) | 0.008 | 3.4  (1.6-7.2) | 0.002 |
|  | High  (API≥100) | 15.0  (9.0-24.1) | <0.001 | 5.1  (2.6-10.0) | <0.001 |
|  |  |  |  |  |  |
